# Probing the Ecology and Climate of the Eocene Southern Ocean With Sand Tiger Sharks *Striatolamia macrota*

**DOI:** 10.1029/2020PA003997

**Published:** 2020-12-08

**Authors:** Sora L. Kim, Sarah S. Zeichner, Albert S. Colman, Howie D. Scher, Jürgen Kriwet, Thomas Mörs, Matthew Huber

**Affiliations:** ^1^ Department of Geophysical Sciences University of Chicago Chicago IL USA; ^2^ Department of Life and Environmental Sciences University of California Merced CA USA; ^3^ Division of Geological and Planetary Sciences California Institute of Technology Pasadena CA USA; ^4^ Department of Earth, Environment, and Planetary Sciences Rice University Houston TX USA; ^5^ Department of Earth, Ocean, and Environment University of South Carolina Columbia SC USA; ^6^ Department of Palaeontology University of Vienna Vienna Austria; ^7^ Department of Palaeobiology Swedish Museum of Natural History Stockholm Sweden; ^8^ Bolin Centre for Climate Research Stockholm University Stockholm Sweden; ^9^ Department of Earth, Atmosphere, and Planetary Sciences Purdue University West Lafayette IN USA

**Keywords:** neodymium isotope analysis, oxygen isotope analysis, paleobiology, paleoclimate, Seymour Island, temperature

## Abstract

Many explanations for Eocene climate change focus on the Southern Ocean—where tectonics influenced oceanic gateways, ocean circulation reduced heat transport, and greenhouse gas declines prompted glaciation. To date, few studies focus on marine vertebrates at high latitudes to discern paleoecological and paleoenvironmental impacts of this climate transition. The Tertiary Eocene La Meseta (TELM) Formation has a rich fossil assemblage to characterize these impacts; *Striatolamia macrota*, an extinct (†) sand tiger shark, is abundant throughout the La Meseta Formation. Body size is often tracked to characterize and integrate across multiple ecological dimensions. †*S. macrota* body size distributions indicate limited changes during TELMs 2–5 based on anterior tooth crown height (*n* = 450, mean = 19.6 ± 6.4 mm). Similarly, environmental conditions remained stable through this period based on δ^18^O_PO4_ values from tooth enameloid (*n* = 42; 21.5 ± 1.6‰), which corresponds to a mean temperature of 22.0 ± 4.0°C. Our preliminary *ε*_Nd_ (*n* = 4) results indicate an early Drake Passage opening with Pacific inputs during TELM 2–3 (45–43 Ma) based on single unit variation with an overall radiogenic trend. Two possible hypotheses to explain these observations are (1) †*S. macrota* modified its migration behavior to ameliorate environmental changes related to the Drake Passage opening, or (2) the local climate change was small and gateway opening had little impact. While we cannot rule out an ecological explanation, a comparison with climate model results suggests that increased CO_2_ produces warm conditions that also parsimoniously explain the observations.

## Key Points

- Sand tiger teeth indicate stable ecology and environment with little change in body size or water temperature during Early‐Middle Eocene
- Eocene Southern Ocean temperatures were likely warmer than previous studies suggest based on geochemical and climate simulation results
- Neodymium isotopes indicate Pacific water inputs in TELM 2, which is the earliest geochemical evidence of the Drake Passage opening

## 1 Introduction

The Eocene marked a period of climate vastly different from today with temperate ecosystems at high latitudes (i.e., Douglas et al., 2014; Eberle & Greenwood, 2012). During this time, climate transitioned from greenhouse to icehouse conditions (Zachos et al., 2008) and impacted the evolutionary history of flora and fauna (Gingerich, 2006; Jocque et al., 2010; Krug et al., 2010; Millar, 1993; Scheibner et al., 2005). Antarctica is central to hypotheses related to this climate shift due to its contiguity to tectonic gateways (i.e., Drake Passage and Tasman Gateway) and amplified temperature effects at high latitudes (Bijl et al., 2013; Borrelli et al., 2014; Kennett, 1977). The interplay between climate and ecology during the Eocene Antarctic is recorded in the remains of temperate marine and terrestrial taxa that indicate different environmental conditions than those today (i.e., Mörs et al., 2020).

**Figure 1:**
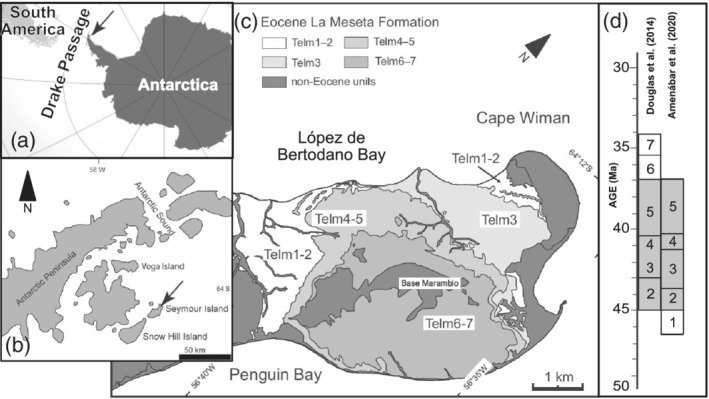
(a) Location of the La Meseta Formation on Seymour Island in relation to South America, Antarctica, and the Drake Passage and (b) in relation to other islands of the Antarctic Peninsula. (c) The relative positioning of TELMs on Seymour Island (from Gaździcki & Majewski, 2012) and (d) proposed TELM chronologies with an indication of absolute age.

The Tertiary Eocene La Meseta (TELM) Fm. on Seymour Island is located east of the Antarctic Peninsula and regarded as the “Rosetta Stone” for Southern Hemisphere evolution because of its excellent preservation of diverse, high latitude flora and fauna that captures the Cenozoic shift from greenhouse to icehouse conditions (Cantrill & Poole, 2012; Figure 1). In addition, the La Meseta Fm. includes a fossil record rich with shark teeth; to date, there are 35 species from 22 families of *Chondrichthyes*, the class including chimeroids, batoids, skates, rays, and sharks identified and described from studies over the past 40 years (see, e.g., Engelbrecht et al., 2017a, 2017b, 2017c, 2017d, 2019; Grande & Eastman, 1991; Kriwet, 2005; Kriwet et al., 2016; Long, 1992; Long & Stilwell, 2000; Marramá et al., 2018; Welton & Zinsmeister, 1980). Here, we delve into the paleoecology of the ancient sand tiger shark, †*Striatolamia macrota* (Agassiz), an extinct (denoted with †), cosmopolitan species found in Eocene nearshore deposits that span from the Arctic (Padilla et al., 2014) to Antarctica (Welton & Zinsmeister, 1980), including midlatitude sites in both hemispheres (Cappetta, 2012). Remains of sand tiger sharks are extremely abundant throughout the La Meseta Fm. (Kriwet et al., 2016). Paleoecological insight to sand tiger sharks from the La Meseta Fm. compliment and expand on the extensive systematic work to date on chondrichthyan fossils (i.e., Engelbrecht et al., 2017a, 2017b, 2017c, 2017d, 2019; Kriwet, 2005; Kriwet et al., 2016; Long, 1992; Long & Stilwell, 2000) and geochemical analyses elucidate paleoceanographic conditions.

**Figure 2:**
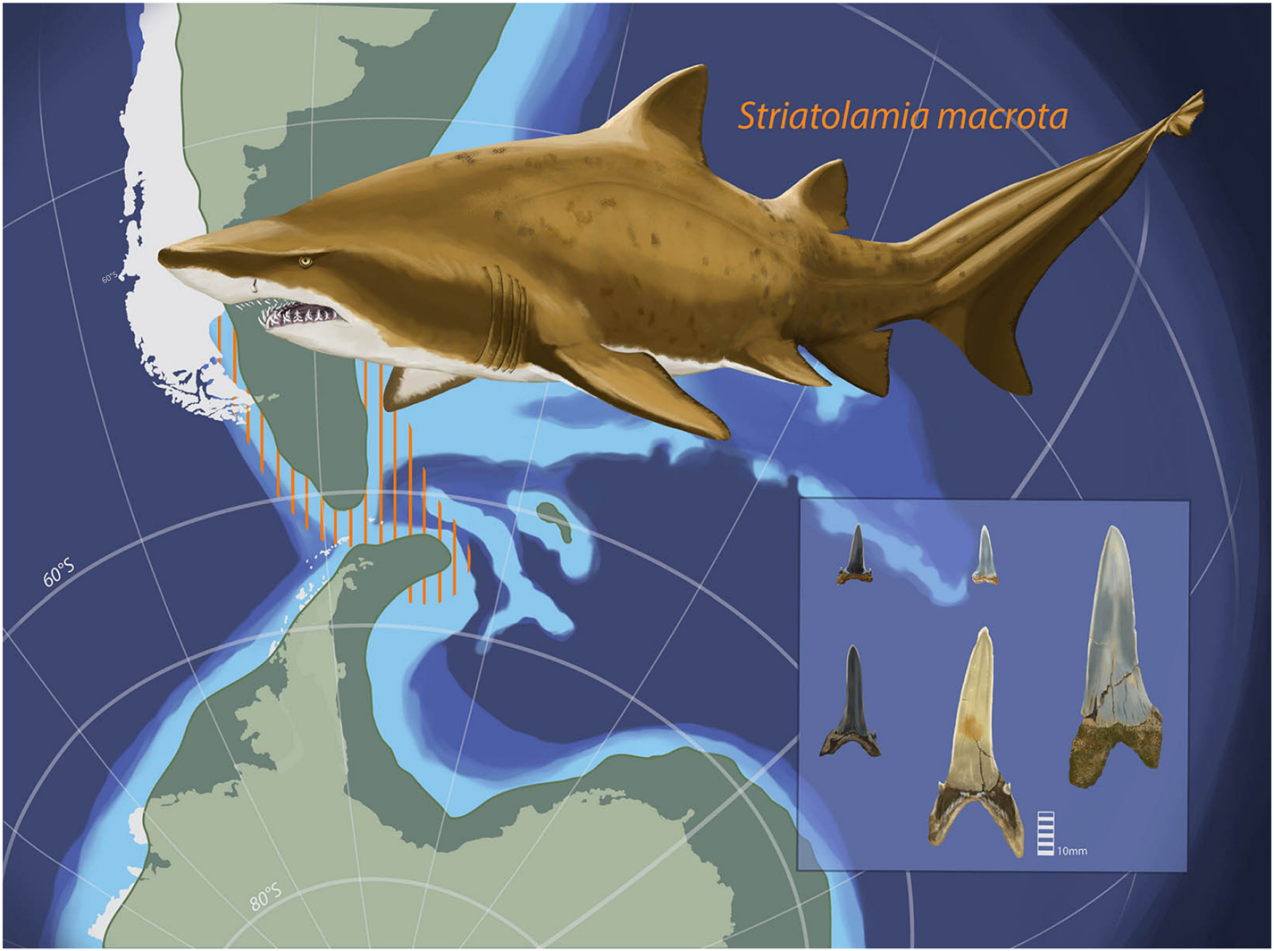
During the Early‐Middle Eocene, †*Striatolamia macrota* inhabited waters off the Antarctic Peninsula and could potentially migrate through a shallow Drake Passage to southern Chile or Argentina. Eocene landmass distribution is shown in green with today's landmass configuration in white; shallow oceans are represented with lighter shades of blue (tectonic and bathymetry reconstructions based on Bohoyo et al., 2019; Lagabrielle et al., 2009; Livermore et al., 2007); vertical orange lines represent shallow marine shelf and proposed area of †*S. macrota* migration to southern South America. Insert includes labial view of five representative anterior teeth, which were used to measure anterior tooth crown height (ATCH) as an indicator of body size. Illustration credit to Christina Spence Morgan.

The extinct sand tiger shark, †*S. macrota*, belongs to the family Odontaspididae in the order Lamniformes and is largely found in Paleocene to Miocene strata (Cappetta, 2012; Kriwet, 2005; Reguero et al., 2012; Figure 2). The modern‐day analog for †*S. macrota* is considered to be *Carcharias taurus* due to similar tooth morphologies and similar inferred habitat preferences based on community assemblage and sedimentology (Case, 1992; Cunningham, 2000; Kim et al., 2014; Kriwet et al., 2016). *Carcharias taurus* is one of three extant sand tiger shark species, and its ecology is well studied off the coasts of Australia (Otway & Ellis, 2011) and eastern United States (Kneebone et al., 2012; Teter et al., 2015). Modern *C. taurus* is known to live in temperate, nearshore waters on continental shelves and migrate annually with a high degree of site fidelity (Kneebone et al., 2012). Additionally, satellite and telemetry tagging studies indicate a preference for surface waters (<90 m) with a limited temperature range (17–24°C), and marine waters, although there is regular movement to low salinity waters (e.g., 25 in Delaware Bay; Kneebone et al., 2012; Teter et al., 2015). In this study, we refer to the extinct Eocene †*S. macrota* and extant *C. taurus* collectively as “sand tiger” sharks.

Here, we seek to explore the interplay between ecology and environment in †*S. macrota* via body size distribution during the Eocene greenhouse—icehouse transition. Shark tooth height directly relates to body size (Shimada, 2004), which is an important ecological characteristic explored in modern and paleontological studies. Body size reflects energy balance, for which there are different demands related to ontogeny, predator pressure, resource availability, and temperature regulation (e.g., Savage et al., 2004; Smith et al., 2010). Studies find that body size plays a role in food web structure (Brown et al., 2004; Cohen et al., 2003; Woodward et al., 2005). While there are no studies that explore the interactive effects of ecology and environment on shark body size, we assume that as ectotherms, their thermal regulation and therefore metabolic rate would be tied to water temperature (Riemer et al., 2018). Further, the response of body size to temperature can alter population dynamics in fish (Lindmark et al., 2018), which has implications related to modern and ancient climate change. Previous studies demonstrated that tooth crown height scales with total body length in sharks as individuals continuously generate new teeth throughout their lifetime (i.e., Shimada, 2002, 2004). Sharks mostly have heterodont dentitions, similar to mammals, and tooth positions can be identified based on morphology then related to body size (Kriwet et al., 2015; Shimada, 1997, 2003, 2004, 2006b, 2019). The abundance of shark teeth in some fossil localities provides an opportunity to determine a body size distribution (i.e., Pimiento & Balk, 2015) that can be associated with environmental conditions (i.e., temperature and salinity) via geochemical analyses (i.e., Kim et al., 2014) or habitat use with morphological context (i.e., Pimiento et al., 2010; Villafaña et al., 2020). Although the Eocene Antarctic was substantially warmer than today and could support temperate taxa, its geographic position resulted in prolonged periods with limited sunlight, reduced temperatures, and seasonal productivity cycles.

Geochemical analyses of fossil shark teeth also yield valuable paleoclimate and paleogeographic data, as teeth record ancient ocean chemistry and temperature within their enameloid. The biological apatite (i.e., bioapatite) of shark enameloid is a fluorapatite (Ca_5_(PO_4_)F). The oxygen isotope composition of bioapatite is dependent on temperature and body water, which is in steady state with environmental water δ^18^O values (Lécuyer et al., 2013; Longinelli & Nuti, 1973; Pucéat et al., 2010). Further, the biologically derived oxygen isotope composition in bioapatite is highly resistant to diagenetic alteration (Vennemann et al., 2001). Previous studies use δ^18^O values of shark enameloid to aid in paleoceanographic reconstructions (Lécuyer et al., 2003; Kocsis et al., 2007; Kim et al., 2014; Pucéat et al., 2003), as the relatively fast mineralization and maturation rate during tooth formation (14–25 days; unpublished data based on captive feeding study by Kim; details of study in Kim, Casper et al., 2012; Kim, Martínez del Rio et al., 2012; Zeichner et al., 2017) preserves environmental conditions. However, shark teeth have a “conveyor belt” replacement system (reviewed in Smith et al., 2013) where tooth formation occurs below the epithelium and teeth progressively move to the front of the jaw over a substantial period of time (~240 days elapsed for leopard shark; Zeichner et al., 2017). Although this rate differs among taxa, the elapsed time between tooth formation and loss means that discarded teeth may not reflect the local environmental conditions for migratory taxa. The oxygen isotope composition of †*S. macrota* teeth from the La Meseta Fm. will elucidate a range of preferred temperatures for this taxon, but it may not reflect the conditions of Seymour Island given the migratory patterns and tooth formation of sand tiger sharks.

As teeth are shed from living sharks and incorporated into the sedimentary record, they incorporate Nd from seawater and early diagenetic fluids (Martin & Scher, 2004). The isotopic composition of Nd in seawater is a function of (1) the crustal age of adjacent lithogenous sediment sources and (2) water mass/ocean current mixing. Further, seawater Nd has a short residence time (500–1,000 years; Tachikawa et al., 2003) relative to ocean mixing scales (~1,500 years). Seymour Island lies along a volcanic margin, so lithogenous inputs are radiogenic (i.e., positive *ε*_Nd_ values, up to + 10). Water masses influencing Seymour Island over the time period under investigation include waters carried by the Weddell Gyre, and after the Middle Eocene, increasing amounts of Pacific water transported through the embryonic Drake Passage (Eagles et al., 2006; Scher & Martin, 2006). The Weddell Gyre carries seawater from the East Antarctic margins, which are influenced by terrains with significantly older crustal ages and thus have less radiogenic compositions (*ε*_Nd_ = −6 to −8) compared to the Antarctic Peninsula volcanics (i.e., Martin & Scher, 2004; Wright et al., 2018). Drake Passage throughflow injects seawater with relatively radiogenic *ε*_Nd_ values (−4 to −6) into the South Atlantic, which is recorded in fossil fish teeth from deep‐sea cores (ODP sites 689 and 1090) and date to the Bartonian (Diester‐Haass & Zahn, 1996; Scher & Martin, 2006; Tripati et al., 2005). Previous *ε*_Nd_ studies focus on fossil fish teeth, but given the similar mineralogical composition of shark teeth (Miake et al., 1991), we expect similarities in Nd uptake. Finally, there is possibility that the *ε*_Nd_ signal of fossil shark teeth in the La Meseta Fm. may indicate an earlier opening of the Drake Passage due to the proximal, shallow depositional environment where Pacific inputs are less diluted.

In this study, we determined body size distribution of †*S. macrota* based on anterior tooth crown height (ATCH) and compared it to modern *C. taurus*. Then, we used oxygen isotope composition of the phosphate from shark tooth enameloid to estimate water temperatures experienced by †*S. macrota*, establish ontogenetic habitat preferences, and compare to previous results of co‐occurring fossil bivalves in the La Meseta Fm. We compared these shark enameloid δ^18^O values and inferred temperatures with results from isotope‐enabled climate model simulations of the Eocene. Finally, we present preliminary Nd isotope results to demonstrate paleoceanographic changes related to the Drake Passage Opening.

## 2 Geologic Setting

The La Meseta Fm. and overlying Submeseta Fm. are located on Seymour Island, 100 km east of the Antarctic Peninsula at 64°17′S, 56°45′W, within the James Ross Basin (Figures 1a and 1b; Dutton et al., 2002; Gaździcki & Majewski, 2012; Ivany et al., 2008; Montes et al., 2013; Sadler, 1988). These two formations consist of a shallow succession of sedimentary marine beds of sandstone, siltstone, and shell, which are stratified into seven numbered lithofacies units referred to as TELM stratigraphic units. The TELM is fault bounded by an angular unconformity at the bottom of the formation and biostratigraphically categorized (Table 1; Long, 1992; Reguero et al., 2012; Sadler, 1988; Stilwell & Zinsmeister, 1992). The Eocene TELM unit and underlying Middle‐Upper Paleocene Cross Valley Fm. together form the Seymour Island Group, which rests on top of the less‐felsic Upper Cretaceous‐lower Paleocene Marambio Group (Gaździcki & Majewski, 2012; Ivany et al., 2008; Marenssi et al., 1998; Montes et al., 2013, 2019; Sadler, 1988; Figure 1c). A previous petrographic study of the La Meseta and Submeseta Formations demonstrated that both have undergone minimal burial and diagenetic alteration (Marenssi et al., 2002).

**Table 1:** Description of TELM Biostratigraphy, Faunal Content, Sedimentology, and Allomembers Compiled From Published Research to Date (Bomfleur et al., 2015; Buono et al., 2016; Friis et al., 2017; Ivany et al., 2008; Kriwet et al., 2016; Marramá et al., 2018; McLoughlin et al., 2016; Montes et al., 2013; Sadler, 1988; Schwarzhans et al., 2017; Stilwell & Zinsmeister, 1992)

TELM	Biostratigraphy	Faunal content	Sedimentology/facies	Marenssi et al. (1998) allomembers	Montes et al. (2013) allomembers	Presence of †*Striatolamia macrota*
5	*Struthiolarella steinmanni* zone	Some *Cucullaea* shell lenses and one layer dominated by the naticid gastropod *Polynices*, which contains marine invertebrates, chondrichthyan and teleost fishes as well as a broad range of terrestrial organisms	Purpled and gray‐green sands and silts. Estuary	Cucullaea I/Cucullaea II	Cucullaea I/Cucullaea II	Abundant
4	*Antarctodarwinella nordenskjoldi* zone	Plethora of *Cucullaea* shells, darwinellid gastropods, and phosphatic shark, ray and bony fish teeth as well as penguin and whale remains	Shell beds with poor stratification. Estuary	Cucullaea I	Cucullaea I	Abundant
3	*Antarctodarwinella ellioti* zone	Dominant in veneroid pelecypods	Buff‐weathering, cross‐bedded sands and silts. Delta plain estuary	Campamento	Acantilados II/Campamento	Abundant
2	*Antarctodarwinella ellioti* zone	Paucity of *Cucullaea* shells	High mud content, well‐preserved stratification that coarsens upwards. Delta front	Acantilados	Acantilados I	Rare
1		*Ostrea* and *Pecten* shells and shell fragments	﻿Red‐brown matrix with two occurrences dominated by either silt or sand. Prodelta?/inner estuarine?	Valle de Las Focas	Valle de Las Focas	Rare

The relative stratigraphic position of TELMs has been agreed upon, but absolute age models of TELMs have changed over time. We adopt the age model from Douglas et al. (2014) and Amenábar et al. (2020) based on their biostratigraphic analysis of the endemic group of dinocyst taxa referred to as “transantarctic fauna.” The lower TELMs (2–3) are determined to be no older than Middle Eocene based on the presence of *Enneadocysta diktyostila* (first occurrence calibrated to Chron C20r at ~45 Ma) and *Arachnodinium antarcticum* and *Hystricosphaerodoim truswelliae* (last occurrences during Chron C18n at ~38 Ma) (Amenábar et al., 2020; Douglas et al., 2014). The Upper La Meseta Fm. (TELM 5) is less well constrained but includes diagnostic dinocyst occurrences of *E. diktyostila*, *Alterbidinium distinctum*, *Brigantedinium spp*., *Lejeunecysta spp*., and *Selenopemphix nephroides* and indicate an age ranging from 41–37 Ma (Amenábar et al., 2020; Douglas et al., 2014). Shark occurances, including †*S. macrota*, are rare in the overlying Submeseta Fm. (TELMs 6–7; Engelbrecht et al., 2017c, 2019; Kriwet et al., 2016), but ages based on ^87^Sr/^86^Sr chemostratigraphy from bivalve carbonates for the Submeseta Fm. units are thought to be consistent with a TELM 6 age of ~41 Ma or younger and the top of TELM 7 lying at the Eocene‐Oligocene boundary (Douglas et al., 2014).

We summarize the biostratigraphy and sedimentology in Table 1 for TELMs 1–5, which contain †*S. macrota* teeth. The Submeseta Fm. also includes TELMs 6 and 7, but these contain little to no sand tiger shark teeth from †*S. macrota* (Kriwet et al., 2016; Long, 1992). In addition to the TELM stratigraphy convention, Marenssi et al. (1998) introduced five erosionally based allomembers for the La Meseta and Submeseta Fms., which roughly correspond to TELMs as reported in Table 1. Further, Montes et al. (2013) divided the Acantilados Allomember into Acantilados I (referred to as Level 32 in some instances) with minor occurrences of *Cucullaea* and Acantilados II (referred to as Level 33 in some instances) with conglomeratic shell lenses cutting into the underlying Acantilados I Allomember. According to Montes et al. (2013), the Acantilados II Allomember represents TELM 3, which results in some uncertainty regarding older samples collected in the Acantillados Allomember sensu (Marenssi et al., 1998) that have been assigned to TELM 2. In the discussion, we give implications to this age uncertainty as it pertains to our geochemical results.

## 3 Methods

### 3.1 Provenance of Material

We focused on †*S. macrota* because these teeth are abundant within the La Meseta Fm. and well studied with a global distribution during the Eocene. Teeth were sampled from collections at the University of California Museum of Paleontology (UCMP; Berkeley, CA, USA), which were described in Long (1992), and the Zinsmeister collection at the Paleontological Research Institute (PRI; Ithaca, NY, USA), which were described in Stilwell and Zinsmeister (1992). The UCMP and PRI specimens were collected during Seymour Island Antarctic expeditions in 1986–1987 and 1989 sponsored by the National Science Foundation. Anterior tooth crown measurements for †*S. macrota* were supplemented by additional teeth from the Paleozoological Collections at the Swedish Museum of Natural History (NRM; Stockholm, Sweden), which are partially described in Kriwet et al. (2016), Engelbrecht et al. (2017a, 2017b, 2017c, 2017d, 2019), and Marramá et al. (2018). NRM specimens were collected by an Argentinian‐Swedish field party as a joint project of the Instituto Antártico Argentino (DNA‐IAA) and the Swedish Polar Research Secretary (SPFS) during the summer campaigns from 2011–2013 on Seymour Island. The three expeditions sampled from different localities within each TELM and do not reference one another.

### 3.2 Body Size Estimates *†S. macrota*

It has been well established that total tooth and crown size of macrophagous lamniforms (taxa with large and differentiated teeth) can be used to infer their total body length, because tooth “growth” is proportional to body growth through tooth replacement (e.g., Shimada, 2006a, 2019; Shimada et al., 2020). The upper first anterior tooth (A1) and lower second anterior tooth (a2) are the tallest teeth in 11 out of 13 macrophagous extant lamniforms (Shimada, 2002), which are generally used for body size inference to reduce the risk of overestimating the total body size (Shimada, 2019). Meanwhile, distinct approaches to calculate the total body length using the tooth crown height are available for most extant (e.g., Shimada, 2003, 2004, 2006b, 2019) and various extinct macrophagous lamniform sharks (e.g., Kriwet et al., 2015; Shimada, 1997, 2007; Shimada et al., 2020) exemplifying the reliability of such body size estimations.

Identification of the exact position of anterior teeth in fossil sharks is hampered by the fact that most elasmobranchs (sharks, rays, and skates) are only represented by their teeth only in the fossil record, due to their poorly mineralized skeletons and the challenge of identifying the exact position of anterior teeth is not always possible in exclusively extinct taxa. Although no articulated dentition of †*S. macrota* has been recovered to date, previous studies have reconstructed its dentition in great detail due to its close dental morphological resemblances to the dentition of the extant sand tiger shark, *C. taurus* (e.g., Cappetta, 2012; Cunningham, 2000; Fieman, 2016). Accordingly, it is easy to identify unambiguously anterior upper and lower teeth in assemblages of †*S. macrota* that can be used to deduce its body size using the crown‐height/body size relationship proposed by Shimada (2004) for the extant sand tiger shark and demonstrated by Fieman (2016).

For this collection, we identified the tooth positions of all teeth of †*S. macrota* from TELMs 2–5 and selected anterior‐most teeth from each collection (TELM). In the following, we used digital calipers to measure the maximum crown width and height (both labial and lingual but report labial measurements only). We analyzed †*S. macrota* teeth from TELMs 2–4 from UCMP; TELMs 2, 3, and 5 from PRI; and TELMs 2–5 from NRM. Previous studies reported that TELM 1 has some bivalve shells present but is sparsely fossiliferous. In addition, there are a few †*S. macrota* teeth known from TELMs 6 and 7 (Kriwet et al., 2016), but their scarcity prevented meaningful data for size distribution analysis. We remeasured every seventh tooth from the UCMP and PRI collections to confirm measurement accuracy and evaluate precision (difference for all for paired samples ±0.3 mm), photographed all teeth, and cataloged individual specimens.

Statistical comparisons of body size distributions within the La Mesta Fm. by TELMs were based on labial ATCH measurements in the statistical software R (R Development Core Team, 2014). We reported ATCH mean, median, maximum (max), and minimum (min) values to represent body size distributions for the entire population by TELM. The equations for A1 (upper) and a1 (lower) presented by Shimada (2004) are slightly different, but the TL estimates we reported are based on an average equation for A1 and a1 position as follows:

(1) TL=−25.7+114×ATCH

where TL and ATCH are both in cm. Body size distributions were compared for TELMs 2–5, as TELM 6 had only four individual teeth from †*S. macrota*. We described body size distributions with skew and kurtosis and determined statistical significance based on the D'Agostino skewness test and Bonett test from the Moments R package. Pairwise comparisons of body size distributions between TELMs 2–5 are based on Kolmogorov‐Smirnov (K‐S) tests. In the discussion, we referred to TL estimates based on ATCH to provide some context of actual body size. We did not make inferences to specific life stages or statistically compare TL between TELMs and therefore expected this approximate TL equation adequate for our discussion purposes.

### 3.3 Isotope Analyses

We selected †*S. macrota* teeth for isotope analysis from several localities within a stratigraphic unit but were limited by the number of available specimens, their preservation state, and the need to maintain collections. The number of teeth measured for isotope analyses is comparable to previous sample sizes for studies that differentiate among species and estimate paleotemperatures based on δ^18^O_PO4_ values of shark enameloid (Amiot et al., 2008; Zacke et al., 2009). The †*S. macrota* teeth analyzed within a TELM for δ^18^O values came from the UCMP and PRI collection. Given the difficulty of stratigraphic control, δ^18^O_PO4_ values from the two collections were grouped together and treated by TELM in this study.

#### 3.3.1 Sampling

We abraded the enameloid with a slow speed dental drill (Foredom TX, Bethel, CT, USA) fitted with a stainless steel, diamond‐coated wheel point (Dremel 7103 5/64‐inch Diamond Wheel Point, Mount Prospect, IL, USA). First, we removed superficial dirt or crust and then powdered the enameloid. All sampling took place under a microscope to ensure exclusion of the inner portion of the tooth where dentin was potentially diagenetically altered.

#### 3.3.2 Oxygen Isotope Analysis

For phosphate oxygen isotope analysis, we followed the rapid, small volume preparation method in Mine et al. (2017). First, we weighed ~1 mg of powdered enameloid and dissolved it in 50 μl of 2‐M HNO_3_ overnight. The next day, Ca^2 +^ was removed as precipitated CaF_2_ from the dissolved bioapatite solution by adding 30 μl of 2.9‐M HF and 50 μl 2‐M NaOH. Then, we pelleted CaF_2_, transferred the supernatant, and repeated the process with a second rinse of 50 μl of 0.1‐M NaF. We added 2‐M HNO_3_ (ca. 30 μl) to adjust solution pH to ~4.5 and added 180 μl of Ag‐ammine solution (1.09‐M NH_4_OH and 0.37‐M AgNO_3_; pH of 5.5–6.5 after addition of Ag‐ammine solution). Finally, we centrifuged samples to pellet the silver phosphate crystals and rinsed the samples five times with deionized water (18.2 MΩ, Barnstead Nanopure, Thermo Fisher Scientific, Waltham, MA, USA). We dried samples overnight at 60°C and weighed all samples in triplicate to 300 ± 100 μg into silver capsules (Costech, Valencia, CA, USA).

The stable isotope composition of oxygen (δ^18^O) was analyzed at the University of Chicago Stable Isotope Ratio facility using a TCEA – Conflo IV – Delta V Plus continuous flow isotope ratio mass spectrometer system (Thermo, Bremen, Germany). We used commercially available Ag_3_PO_4_ (>99% purity) from Strem Chemicals (*n* = 8 per run; Newburyport, MA, USA) and Elemental Microanalysis (*n* = 25 per run; Okehampton, UK) with δ^18^O_PO4_ values of 8.2 ± 0.2‰ and 21.9 ± 0.2‰, respectively, as in‐house reference standards. These isotope compositions represent mean values from multiple calibrations against fluorination values reported for YR‐1, YR‐2, YR‐3, and TU standards (Vennemann et al., 2002). Reference standards were used to monitor for mass linearity and run drift as well as normalization corrections. Benzoic acid was analyzed at the beginning of each run as an oxygen yield standard. Oxygen isotope compositions of phosphate oxygen are reported on the Vienna Standard Mean Ocean Water (V‐SMOW) scale. All reported values are mean ± 1σ from triplicate analysis, unless specified as standard error (s.e.m.). Average and standard deviation values reported for a TELM or the entire dataset have error based on individual samples analyzed in triplicate.

#### 3.3.3 Neodymium Isotope Analysis

For neodymium isotope analysis, we analyzed one tooth each from TELMs 2–5, to evaluate changes in ε_Nd_ values of the La Meseta Fm. over time. Neodymium isotope ratios were analyzed at University of South Carolina Center for Elemental Spectrometry (CEMS) and processed through the single column method (Scher & Delaney, 2010) with column lengths doubled to improve separation of samarium. All measurements were made on a Neptune multiple collector inductively coupled plasma mass spectrometer (MC‐ICP‐MS, Thermo Scientific) with an Apex HF or Apex Q as the introduction system. A standard Ni sample cone and Ni X‐skimmer cone were used. All Nd isotope measurements were made in static mode, and each run consisted of 50 cycles of 8 s. Masses 142–150 were collected in cups L1 through H4, with mass 146 in the center cup. Prior to each analysis, all masses were measured for 10 8‐s cycles for blank subtractions. Blank corrections were negligible owing to effective washout of the previous sample. The Nd isotope standard, JNd1‐1, was run after every fourth sample within the run to monitor instrumental uncertainty (0.2 *ε*_Nd_; Tanaka et al., 2000) and to normalize ^143^Nd/^144^Nd values to 0.512115 (Tanaka et al., 2000). Nd isotopes were measured while monitoring masses 147 and 149 (Sm) allowing for interference corrections on 144, 148, and 150 (Nd). These corrections are negligible because of very small ^147^Sm and ^149^Sm intensities (0.01% of signal). Instrumental mass discrimination was corrected relative to ^146^Nd/^144^Nd = 0.7219 using an exponential law. We calculate *ε*_Nd_ values normalized to the value for the chondritic uniform reservoir (CHUR; 0.512638) and report *ε*_Nd_ ± 2 s.e.m.

### 3.4 Paleotemperature Estimates

We applied the Pucéat et al. (2010) phosphate oxygen isotope paleothermometer to the δ^18^O values from the †*S. macrota* enameloid:

(2) $$T=124.6\;\left(\pm 9.5\right)-4.52\;\left(\pm 0.41\right)\times \left(\delta^{18}O_{\mathrm{PO}4}-\delta^{18}O_{H20}\right)$$

where *T* is the temperature of the water in °C when the enameloid mineralized. We chose to apply the Pucéat et al. (2010) phosphate oxygen paleothermometer because it is (1) based on a tightly controlled experiment with captive fish in aquaria and biomarkers that indicated timing of tooth mineralization; (2) consistent with experimental results that show an offset between dissolved vs. mineral PO_4_ (Chang & Blake, 2015); and (3) corresponds to patterns recorded in mammalian teeth (Green et al., 2018). For δ^18^O_H2O_ values, we relied on the combined approach by Douglas et al. (2014) where bivalves from the La Meseta Fm. were analyzed for both “clumped” composition (Δ_47_) to constrain temperature and bulk carbonate composition (δ^18^O values) to estimate δ^18^O_H2O_ values. We applied δ^18^O_H2O_ values of −1.17‰, −1.11‰, −1.06‰, and −1.00‰ for TELMs 2–5, respectively, based on Douglas et al. (2014) (equation from Douglas et al. Fig. S2) but acknowledge that there is uncertainty in δ^18^O_H2O_ values. We tested the sensitivity of various paleothermometers (i.e., Chang & Blake, 2015; Kim et al., 2007; Lécuyer et al., 2013; Longinelli & Nuti, 1973; Pucéat et al., 2010) and found that the variation within the †*S. macrota* teeth sampled is greater than the variation produced across all paleothermometers. Additionally, we compared different δ^18^O_H2O_ values (i.e., Douglas et al., 2014; Ivany et al., 2008; Lear et al., 2000; Zachos et al., 1994) estimated for high latitudes in the Eocene Southern Hemisphere and found the magnitude of variation from †*S. macrota* teeth the most influential. We did not propagate error with the paleothermometer equation, as the variation of δ^18^O_PO4_ values from teeth within each TELM was so large. The mean and standard deviation in temperature we report for each TELM and the La Meseta Fm. are based on the individual specimen estimates (measured in triplicate). We treated all data from each TELM collectively without stratigraphic differentiation.

### 3.5 Model‐Data Comparison and Synthesis

We developed an alternative framework to interpret δ^18^O_PO4_ values that is independent of geochemical analyses and based on the results of a global climate model simulation. We incorporated the results of the water isotope‐enabled simulations using iCESM1.2 (Brady et al., 2019) carried out by Zhu et al. (2020), which extended the study of Zhu et al. (2019). These simulations, built upon the boundary conditions of Herold et al. (2014), following the Deemip protocol (Lunt et al., 2017), are uniquely capable of matching existing surface temperature data in the Early Eocene (Hollis et al., 2019; Zhu et al., 2019). For this comparison, we used output from Zhu et al.'s (2020) fully equilibrated simulations at 3× and 6× preindustrial CO_2_ levels to predict surface temperature and δ^18^O_H2O_ values. As described in more detail in Zhu et al. (2020), these simulations were carried out for 2,600 and 2,000 years, with trends in global mean surface temperature of −0.34° and −0.03° and trends in global mean sea‐surface δ^18^O_H2O_ trends of 0.04, −0.06‰/1,000 years (for the 3× and 6× CO_2_ cases respectively). These predictions allowed us to establish an independent and self‐consistent check on the relationship between temperature and δ^18^O_PO4_ values by inverting Equation 2 and comparing the resulting values with those measured from shark teeth enameloid. We referred to estimates from these model simulations as δ^18^O_PO4_^*^ values.

## 4 Results

### 4.1 La Meseta Fm. †*S. macrota* Size Distribution

**Figure 3:**
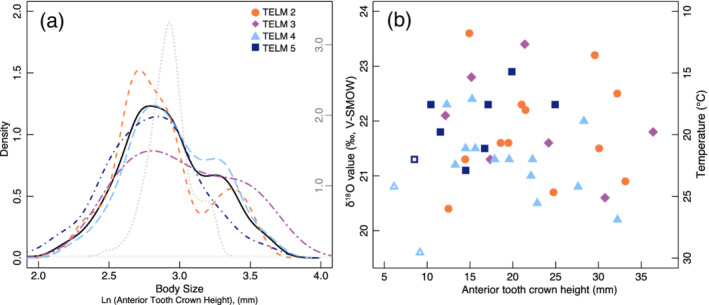
Fossil shark teeth were measured for anterior tooth crown height (ATCH), which correspond to total length. (a) The La Meseta Fm. body size distributions are shown as probability density functions and similarity among TELMs indicate ecological stasis. The gray dotted line represents the modern Delaware Bay population and corresponds with the right *y*‐axis. (b) Body size and δ^18^O values lack correspondence and suggest no differences in temperature preference with age. Open symbols represent teeth that are likely from the symphyseal position rather than an anterior position.

ATCH distributions provided context to evaluate changes in †*S. macrota* body size through time at the La Meseta Fm. (Dataset DS1; Figure 2). The measured mean ATCH of †*S. macrota* (±1σ) was 19.6 ± 6.4 mm (*n* = 450) and median = 18.0 mm with minimum ATCH of 10.0 mm and maximum anterior tooth height of 41.0 mm (Figure 3a). The ATCH of †*S. macrota* in TELM 2 ranges from 10.5–33.9 mm (mean = 17.0 mm), that in TELM 3 from 12.1 to 36.4 mm (mean = 19.0 mm), that in TELM 4 from 10.0 to 41.0 mm (mean = 18.5 mm), and that in TELM 5 from 11.0 to 38.0 mm (mean = 17.3 mm). Employing the formula provided by Shimada (2004) for the extant sand tiger shark *C. taurus*, the body size ranges from 94 to 361 cm in TELM 2, from 112 to 389 cm in TELM 3, from 88 to 442 cm in TELM 4 and from 100 to 408 cm in TELM 5. Consequently, the smallest (88 cm) and largest (442 cm) specimen occur in TELM 4, from which also the largest sample was obtained. The ATCH distributions, nevertheless, did not significantly differ among TELMs (Figure 3a; Table 2).

**Table 2:** Summary of Anterior Tooth Crown Height and Stable Isotope Composition Data Reported in This Study

	All	TELM 2	TELM 3	TELM 4	TELM 5
ATCH, *n*	450	51	13	277	109
Mean ± 1σ (mm)	19.6 ± 6.4	18.7 ± 6.2	21.8 ± 8.5	20.2 ± 6.5	18.4 ± 5.5
Median (mm)	18.0	17.0	19.0	18.5	17.3
Range (mm)	10.0–41.0	10.5–33.9	12.1–36.4	10.0–41.0	11.0–38.0
D'Angostino test	Skew = 0.93, *z* = 7.08, *p* < < < 0.0001	Skew = 1.03, z = 2.93, *p* = 0.003347	Skew = 0.57, *z* = 1.08, *p* = 0.2786	Skew = 0.81, *z* = 5.02, *p* < < < 0.0001	Skew = 1.22, *z* = 4.53, *p* < < < 0.0001
Kurtosis‐Bonett test	*τ* = 5.08, *z* = −0.20, *p* = 0.845	*τ* = 4.80, *z* = 0.52, *p* = 0.601	*τ* = 7.03, z = −1.18, *p* = 0.238	*τ* = 5.32, *z* = −1.32, *p* = 0.186	*τ* = 4.20, *z* = 1.54, *p* = 0.122
δ^18^O, *n*	42	12	7	15	8
Mean δ^18^O ± 1σ (‰)	21.6 ± 1.6	21.8 ± 1.2	21.9 ± 0.5	21.2 ± 0.8	22.0 ± 0.6
δ^18^O range (‰)	19.6–23.6	20.4–23.6	20.6–23.4	19.6–22.4	21.1–22.9
Inferred temperature^a^	13–31	13–27	14–26	18–31	16–25
*ε*_Nd_ ^b^	—	–6.1 ± 0.2	−5.5 ± 0.2	−5.1 ± 0.1	−5.3 ± 0.1

^a^ Temperature estimates based on δ^18^O_H2O_ values per TELM from Douglas et al. (2014) and Pucéat et al. (2010) paleothermometer.

^b^ Error is analytical s.e.m. reported for analysis.

The entire sampled population exhibited statistically significant skew (D'Angostino test skew = 0.93, *z* = 7.08, *p* < < < 0.0001) but not kurtosis (Bonett test *τ* = 5.08, *z* = −0.20, *p* = 0.845). The ATCH distributions from TELMs 2, 4, and 5 also exhibited statistically significant skew but not kurtosis (see Table 2 and Figure 3a). The ATCH distributions did not significantly differ among TELMs (Figure 3a; Table 2). †*S. macrota* teeth recovered from TELM 6 were not included in size distribution analysis because of the low total count (*n* = 4).

### 4.2 δ^18^O Values From †*S. macrota*

**Figure 4:**
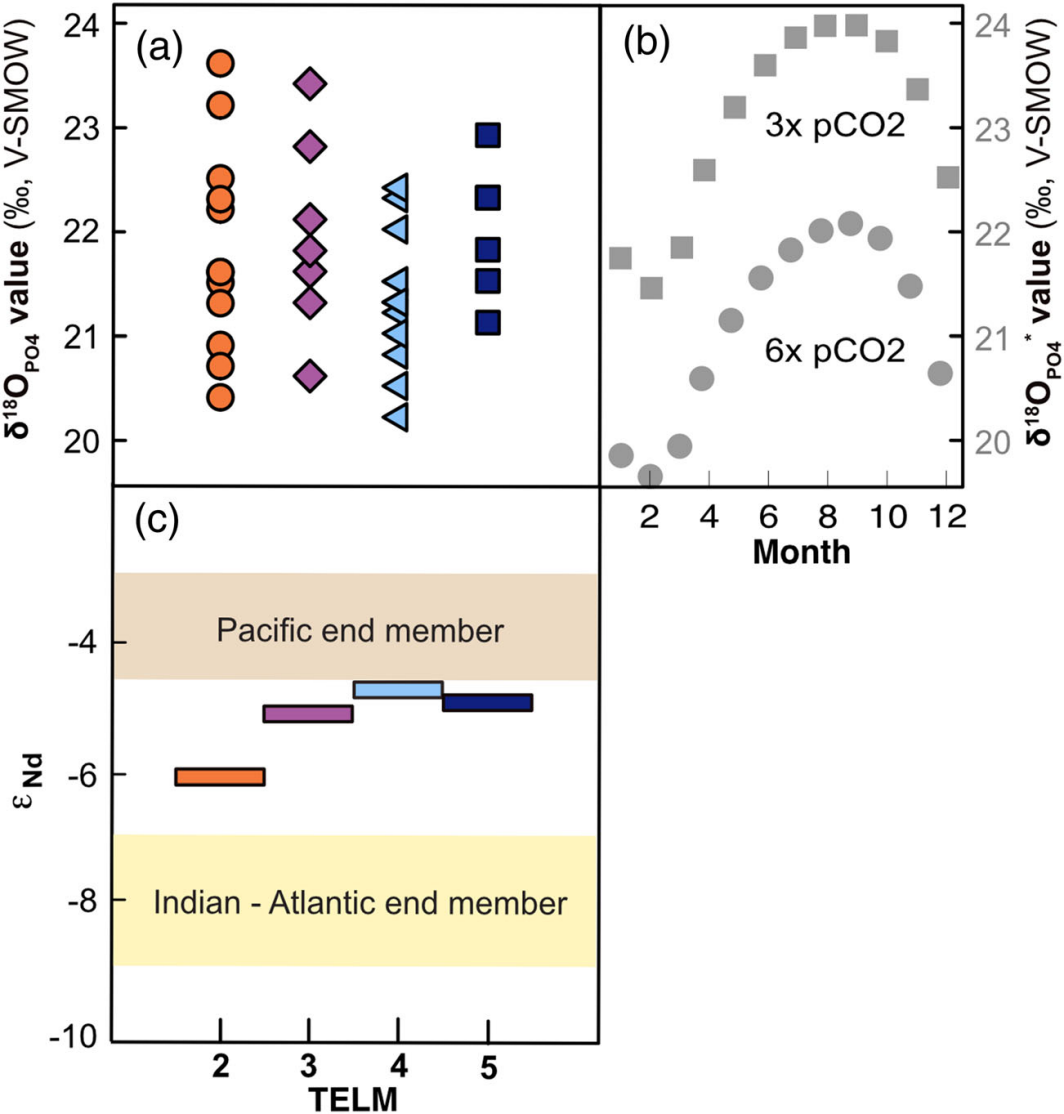
(a) The δ^18^O values from enameloid phosphate of †*S. macrota* teeth collected from La Meseta Fm. grouped by TELM. (b) Forward modeling with isotope‐enabled climate simulation results (Zhu et al., 2020) estimate δ^18^O_PO4_* values of similar range and variability when CO_2_ is 3× and 6× preindustrial levels. (c) There is evidence of a Drake Passage Opening as early as TELM 2 based on neodymium isotope results with Indian‐Atlantic and Pacific endmembers indicated (*n* = 1 per TELM).

†*Striatolamia macrota* teeth from La Meseta Fm. have a mean δ^18^O_PO4_ value ± 1σ of 21.6‰ ± 1.6‰ (*n* = 42; 1σ includes standard deviation of triplicate sample analysis and across all teeth measured within a TELM). Of the †*S. macrota* teeth analyzed for isotopic composition, the mean ± 1σ ATCH is 19.8 ± 7.7 mm (*n* = 42) and ranged 10.4–36.4 mm, which spans this study's range measured for the population of †*S. macrota* from the La Meseta Fm. (Figure 3b). We also included isotopic results from three teeth initially classified as anterior, but then on further inspection determined to be symphysial teeth in our analysis (open symbols in Figure 3b). We found no correlation between ATCH and δ^18^O_PO4_ values (Figure 3b), which suggested no substantial changes in environmental conditions (i.e., salinity and/or temperature) with ontogeny. The average δ^18^O_PO4_ ± 1σ values by TELM are as follows: TELM 2 = 21.8 ± 1.2‰ (*n* = 12), TELM 3 = 21.9 ± 0.5‰ (*n* = 7), TELM 4 = 21.2 ± 0.8‰ (*n* = 15), and TELM 5 = 21.9 ± 0.6‰ (*n* = 8) (Figure 4a; Table 2; symphyseal teeth not included). Sample IDs, collection names, δ^18^O_PO4_ ± 1σ values, and temperature estimates are reported in Table S1.

We used δ^18^O_PO4_ values to estimate average paleotemperature per TELM (±1σ, uncertainty determined from the mean temperature estimate based on individual teeth δ^18^O_PO4_ values), which are as follows: TELM 2 = 20.6 ± 4.5°C (*n* = 12), TELM 3 = 20.5 ± 4.2°C (*n* = 7), TELM 4 = 24.0 ± 3.4°C (*n* = 15), and TELM 5 = 20.9 ± 3.1°C (*n* = 8). Our temperature estimates were based on estimated δ^18^O values for seawater, which varied 0.17‰, as reported in Douglas et al. (2014) and detailed in section 3 (Table 1 and Figure 4a).

### 4.3 Estimates of δ^18^O_PO4_^*^ Values Based on Climate Model Results

**Figure 5:**
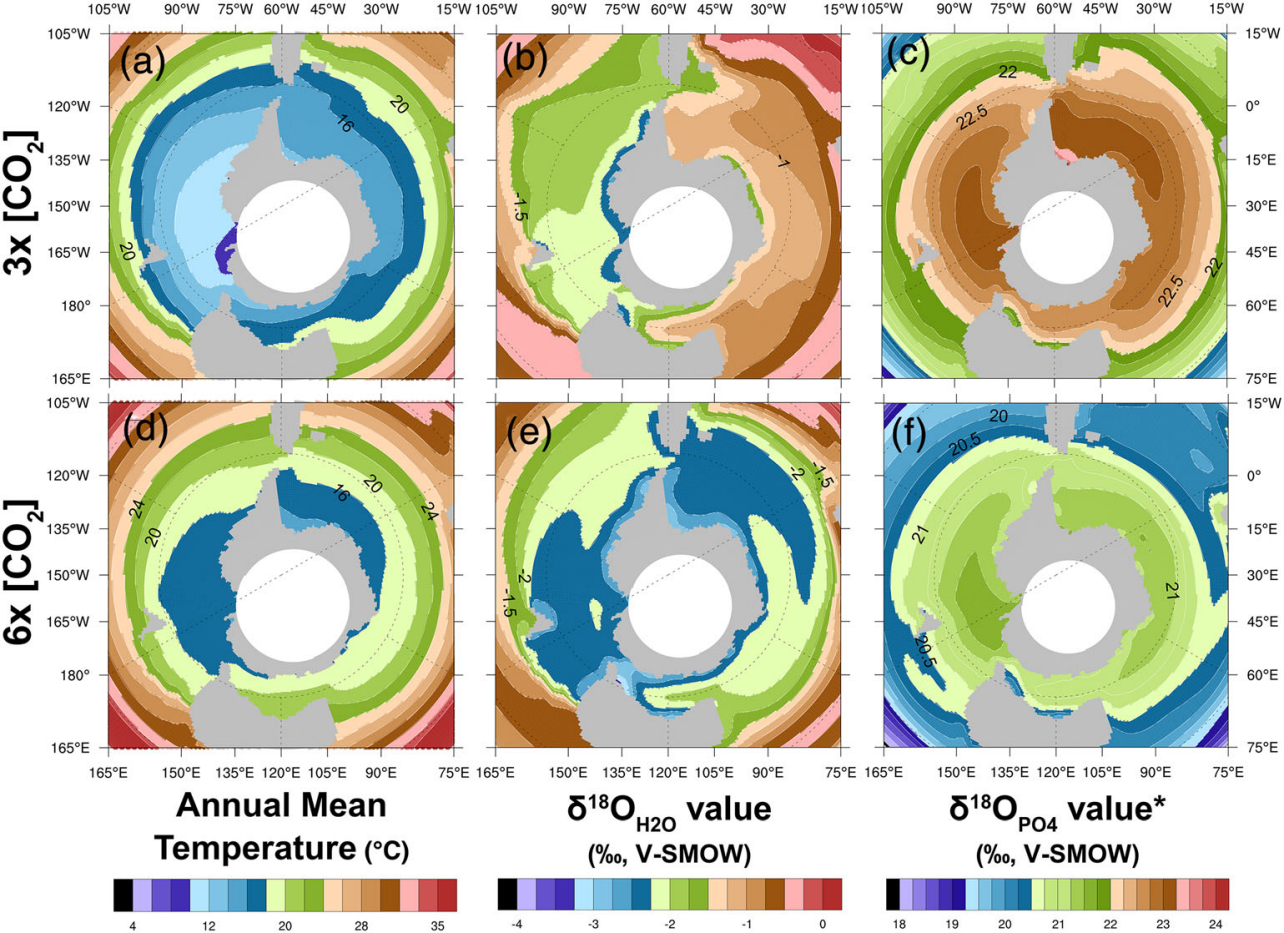
An isotope‐enabled global climate simulation for the Early‐Middle Eocene provides insight to (a and d) temperature and (b and e) δ^18^O_H2O_ values based on 3× (top row) and 6× (bottom row) preindustrial CO_2_ levels (Zhu et al., 2020). These results can be used in a forward model (Equation 2) to predict (c and f) expected δ^18^O_PO4_^*^ values of shark tooth enameloid.

Our estimated δ^18^O_PO4_^*^ values capture the seasonal variability in surface temperature and δ^18^O_H2O_ values from Zhu et al. (2020). In the 3× preindustrial CO_2_ levels, we predicted the lowest δ^18^O_PO4_^*^ values for the month of February with an average of 21.9‰ (range of 21.8–22.0‰) and the highest δ^18^O_PO4_^*^ values for the month of September with an average of 23.9‰ (range of 23.8–24.0‰) (Figure 4b). The Zhu et al. (2020) simulation for 6× preindustrial CO_2_ levels resulted in warmer temperatures and lower δ^18^O_H2O_ values, which produced the expected pattern in δ^18^O_PO4_^*^ values; the lowest δ^18^O_PO4_^*^ values were again in February with an average of 19.5‰ (range of 19.4–19.6‰) and highest δ^18^O_PO4_^*^ values in September with an average of 22.3‰ (range of 22.2–22.3‰) (Figures 5c and 5d).

### 4.4 *ε*_Nd_ Values

*ε*_Nd_ values varied approximately 1‰ between TELMs, with an overall increasing trend. TELM 2 had the least radiogenic *ε*_Nd_ value, and all †*S. macrota* neodymium isotopic compositions were consistent with neodymium signals between the Eocene Pacific and Atlantic oceans (Scher & Martin, 2006). For TELM 2 *ε*_Nd_ = −6.1 ± 0.2, TELM 3 *ε*_Nd_ = −5.5 ± 0.2, TELM 4 *ε*_Nd_ = −5.1 ± 0.1, and TELM 5 ε_Nd_ = −5.3 ± 0.1 (*n* = 1 per TELM; 1σ is based on analytical uncertainty) (Figure 4c). Reported uncertainties for *ε*_Nd_ values = 2 s.e.m, which were less than instrumental uncertainty (0.2).

## 5 Discussion

### 5.1 Paleoecology: Body Size

Our results indicate no substantial ecological change for †*S. macrota* through the La Meseta Fm., as represented by body size distributions extrapolated from ATCH measurements. The sample size of †*S. macrota* anterior teeth within the La Meseta Fm. (*n* = 450) allows robust body size estimates and distributions. The ATCH for the La Meseta Fm. †*S. macrota* teeth reflects a TL range of 88–442 cm, which is greater than that known for modern *C. taurus*. Shimada et al. (2020) (Tab. 3) reconstructed a maximum body size of 495 cm for †*Striatolamia* (presumably †*S. macrota)*. The documented *C. taurus* size range includes parturition TL = 91–105 cm (reviewed in Gilmore, 1993) and adult TL = ~300–320 cm based on age growth studies (Branstetter & Musick, 1994; Goldman et al., 2006). The range of estimated body sizes for †*S. macrota* within each TELM indicates that each assemblage consists of young‐of‐the‐year (YOY), juveniles, subadults, and even mature adults, suggesting an absence of segregation by body size. A similar pattern also was reported for the extant sand tiger shark, *C. taurus* (Dicken et al., 2006, 2007, 2008).

To evaluate if the population of †*S. macrota* in the La Meseta Fm. represent a subset of a population, we transformed TL measurements for modern *C. taurus* to ATCH and compared to those measured in this study. A well‐studied *C. taurus* population in Delaware Bay has TLs ranging 89–266 cm (Goldman et al., 2006; Haulsee et al., 2018). The associated ATCH for this Delaware Bay population is 10–26 mm (based on Equation 1), which is a smaller ATCH range than †*S. macrota* from the La Meseta Fm. (Figure 3a; gray dotted line). The discrepancy in extant vs. extinct total body length could be due to differences in the correlation between total length versus ATCH for extant and extinct sand tiger sharks since the specimens featured in Shimada's regression study span TL 100–300 cm (Shimada, 2004). Another possibility is the misclassification of tooth position when sorting †*S. macrota* material (i.e., some A2 and a2 teeth were included in the sample set). In comparing †*S. macrota* and extant *C. taurus* body size distributions, it may be worth mentioning that these distributions have similar shapes; there is a bimodal distribution with the bulk of density in the lower mode (Figure 3a). Additionally, the median ATCH estimated for the Delaware Bay population (based on Equation 1) is 18.7 mm, which is similar to the median ATCH for each TELM and the combined †*S. macrota* population measured from the La Meseta Fm. Life stages (i.e., YOY, juvenile, and adult) of modern *C. taurus* have variable TL estimates at different locations (Kneebone et al., 2014; Lucifora et al., 2009), but the corresponding ATCH for these life stages is within the range measured for †*S. macrota* from La Meseta Fm. Based on the large range and median values of ATCH in this study, we conclude that the †*S. macrota* teeth from the La Meseta Fm. represent the body size range and all life stages (YOY, juveniles, subadults, and even mature adults) of the extinct sand tiger shark throughout the fossil‐bearing section as well as within each TELM, a pattern that also was reported for the extant sand tiger shark, *C. taurus* (Dicken et al., 2006, 2007, 2008). We therefore assume that each TELM, as well as the entire La Meseta Fm. assemblage, represents a significant subsample of the entire population. Finally, the La Meseta Fm. †*S. macrota* teeth suggest no ontogenetic patterns in habitat preference based on ATCH and δ^18^O values (Figure 3b).

This relatively large range in body size indicates a productive ecosystem in the near shore environment off the Antarctic Peninsula at Seymour Island. Further the stability of †*S. macrota* body size distribution throughout TELMs 2–5 suggests continued suitable habitat and resources, such as food availability, for these top predators. It has been shown that larger species generally have better niche and optimal diet positions as well as a larger range of prey when the food web is complex (Williams et al., 2010). We can assume that the food web at Seymour Island during the Eocene was complex and stable based on the highly diverse fauna (Reguero et al., 2012), including elasmobranch (Engelbrecht et al., 2017a, 2017b; Kriwet, 2005; Kriwet et al., 2016) and also teleost fauna (Přikryl & Vodrážka, 2012; Schwarzhans et al., 2017). Larger body size increases the possible prey range of *S. macrota*, which makes it a keystone predator in the Eocene Antarctic environment, even though the largest individuals from Seymour Island are slightly smaller than the largest calculated specimen recorded by Shimada et al. (2020).

### 5.2 Paleoenvironmental Reconstruction: Temperature

Oxygen isotope measurements are a valuable geochemical technique, often used to reconstruct ocean paleotemperature. For temperature estimates from oxygen isotopes to be accurate, paleothermometers assume environmental water δ^18^O values, which can vary with salinity and latitude. The measured mean δ^18^O_PO4_ values of our †*S. macrota* teeth fluctuates ~0.7‰ among TELMs 2–5, which indicates little secular trend in water conditions through this time. However, the assemblage of teeth analyzed for each TELM reveals a considerable amount of variation with 1σ = 0.5–1.2‰ (i.e., ~3–8.5°C, Figure 3b). This variability is distinct from analytical uncertainty, which is determined by preparation standards and reference materials, but rather represents an environmental signal during the sharks' lifetime.

Paleotemperature estimates from oxygen isotope measurements of †*S. macrota* were warmer than previous estimates based isotopic analyses of co‐occurring bivalve shells (i.e., Douglas et al., 2014; Ivany et al., 2008). The δ^18^O_PO4_ variation measured from †*S. macrota* teeth was consistent with seasonal variation captured from serial sampling of co‐occurring bivalves from the La Meseta Fm. (Judd et al., 2019) but did not capture the cooling trend over time from previous studies (Douglas et al., 2014; Ivany et al., 2008). For example, the carbonate of co‐occurring bivalves record temperatures over time from ~17°C (TELM 2) to ~10°C (TELMs 6 and 7; Douglas et al., 2014; Ivany et al., 2008; Judd et al., 2019). We applied a recent calibration of the Δ_47_ paleothermometer to prior bivalve Δ_47_ measurements, which yielded cooler temperature estimates from those previously published (Table S2; *T*avg, Kelson et al., 2017 = 7.7 ± 2.9°C vs. *T*avg, Douglas et al., 2014 = 13.6 ± 2.3°C, disregarding value from LMF cement; Kelson et al., 2017). Further, when this revised temperature from the Δ_47_ calibration of Kelson et al. (2017) is applied to the Grossman and Ku (1986) paleothermometer, the estimated δ^18^O_H2O_ value for bivalve shells reported in Douglas et al. (2014) is –2.8 ± 1.0‰, which is lower than values initially reported (–1.2 ± 0.9‰) and similar to results from the isotope‐enabled climate simulations for 3× and 6× CO_2_ of Zhu et al. (2020). However, we note that these revised temperature and δ^18^O_H2O_ estimates based on the Δ_47_ calibration of Kelson et al. (2017) are an approximation as we do not account for internal carbonate standard differences, acid digestion fractionation (Δ^*^_25−X_), or ^17^O corrections, which are important factors in clumped isotope paleothermometer applications (Olack & Colman, 2019; Petersen et al., 2019; Schauer et al., 2016). Given this difficulty in revisiting past Δ_47_ results, the temperatures we report for measured δ^18^O_PO4_ values from †*S. macrota* teeth use the δ^18^O_H2O_ values previously published in Douglas et al. (2014). To date, all carbonate analyses of bivalve shells estimate substantially cooler temperatures than those captured by phosphate oxygen analysis of †*S. macrota* teeth. We propose that this offset reflects the relatively shallow, surface waters inhabited by sand tiger sharks, which is predominantly <90 m for modern *C. taurus* (Kneebone et al., 2014; Teter et al., 2015). The warm temperatures indicated from †*S. macrota* teeth across TELMs (TELM 2 = 20.6 ± 4.5°C, TELM 3 = 20.5 ± 4.2°C, TELM 4 = 24.0 ± 3.4°C, and TELM 5 = 20.9 ± 3.1°C; Figure 3b) are consistent with the temperatures inhabited by modern *C. taurus* (17–24°C; Kneebone et al., 2014; Otway & Ellis, 2011; Teter et al., 2015).

In comparing temperature estimates from sharks versus bivalves, time represented and therefore seasonality effects are important to consider. The two bivalve species measured in these studies preferentially grow and integrate temperature within alternate seasons (i.e., *Cucullaea* grows mainly in winter, while *Eurhomalaea* captures most of the seasonal cycle), and thus their combined signal is thought to represent annual temperature variation over the Eocene for shallow nearshore waters near Seymour Island (Douglas et al., 2014; Ivany et al., 2008; Judd et al., 2019). However, the discrepancy in temperature estimates from bivalve shells and shark teeth suggest that bivalve growth and shell accretion is likely minimal or halted during peak warm month temperatures. This explanation is supported by analyzing seasonally clipped results from the isotope‐enabled climate simulations for 3× and 6× CO_2_ of Zhu et al. (2020), during the cool season of May–October, mean temperature estimates are 10–12°C at Seymour Island, which is in contrast to the results of increased zonal heterogeneity from Douglas et al. (2014).

Diagenesis was not explored as an explanation for isotopic results because shark teeth are highly resistant to alteration (Vennemann et al., 2001). Further, a mineralogical study of underlying La Meseta Fm. sediments concluded minimal burial (<1 km) and heating (<90°C) (Pirrie et al., 1998) and biogenic carbonates analyzed to date yield no indication of alteration (Douglas et al., 2014; Ivany et al., 2008). Two potential hypotheses to reconcile our observed ecological stasis of †*S. macrota* and paleotemperature results with previous studies are as follows: (1) modification of seasonal migration behavior or (2) minimal environmental change in the pelagic waters of Seymour Island.

### 5.3 Explanations for Ecological and Environmental Stasis in †*S. macrota*

There is a possibility that the ecological and environmental stasis of †*S. macrota* was not a product of stable conditions at Seymour Island, but rather from seasonal migrations to track thermal isoclines. We evaluate the possibility of seasonal migration and warmer than previously thought temperatures for an Eocene Seymour Island. The seasonal migration of modern *C. taurus* closely tracks temperature (Kneebone et al., 2014; Otway & Ellis, 2011; Teter et al., 2015), which may be a conserved trait among sand tiger sharks. If temperatures at Seymour Island gradually decreased, as recorded by fossil bivalves (Douglas et al., 2014; Ivany et al., 2008), it is possible that †*S. macrota* modified its migration behavior to ameliorate impacts of this environmental change. Seasonal migrations would influence δ^18^O_PO4_ values (Figure 4a) of †*S. macrota* teeth. There is a delay between tooth mineralization, which occurs at the back of the jaw, to the functional position in the first series. Thus, teeth lost at Seymour Island may reflect a larger spatial signal. While the timing of tooth movement through the conveyor system varies among taxa, it is quantified for *Triakis semifasciata* as 240–265 days (Zeichner et al., 2017). The most likely areas to span †*S. macrota* migration are waters off the coasts of southern Chile or Argentina where estimated sea surface temperatures were 14–23°C (Figure 2; Douglas et al., 2014; Zhu et al., 2020). The suitability of this region for †*S. macrota* is also supported by fossil evidence in Eocene‐age assemblages of the Río Turbio and Loreto Fm. of southern Chile (Otero & Soto‐Acuña, 2015). In this explanation, we hypothesize that as oceanographic conditions changed with the Drake Passage and Tasman Gateway opening, †*S. macrota* could have initiated or lengthened the duration of seasonal migrations north to access temperate water. Tectonic models suggest that Seymour Island and the nearby Antarctic Peninsula had a shallow continental ocean shelf before and during early stages of the Drake Passage Opening (Figure 2; Livermore et al., 2007; Lagabrielle et al., 2009), which is similar to the environment and distance of the modern *C. taurus* seasonal migration (Kneebone et al., 2014; Otway & Ellis, 2011; Teter et al., 2015). If migration is a conserved trait among sand tiger sharks, this system could serve as an example of ecological plasticity in sharks to mitigate climate change effects.

The environmental water temperatures from †*S. macrota* enameloid are substantially warmer than those proposed from other indicators from high‐latitude, Southern Hemisphere waters as well as regional and global climate simulations (Table S3). The ecological and environmental stasis we observe from †*S. macrota* teeth is unsurprising given that climate model results have not produced large changes for gateway opening of this scale (Goldner et al., 2014). Climate simulations are strongly dependent on input CO_2_ concentration, paleogeography, and ocean circulation (Table S4; Kennedy et al., 2015; Ladant et al., 2014; Lunt et al., 2012; Tindall et al., 2010; Zhu et al., 2019), but the temperature estimates based on δ^18^O values from †*S. macrota* in this study are the warmest reported from a high latitude locality, such as Seymour Island. The most similar environmental water temperatures to those presented in this study are from pelagic foraminifera δ^18^O values and TEX^86^ from the high‐latitude South Pacific Ocean (Bijl et al., 2009; Hollis et al., 2009; see Table S4), which are considered biased toward seasonal high temperatures (Hollis et al., 2012). However, we propose that high‐latitude, Southern Hemisphere waters were warmer than previously considered, as indicated by the δ^18^O_PO4_ values from †*S. macrota* in this study and recent isotope‐enabled climate simulations for 3× and 6× CO_2_ of Zhu et al. (2020).

A forward model based on the combined output of temperature and δ^18^O_H2O_ values from Zhu et al. (2020) provides a proxy independent estimate for comparison to δ^18^O_PO4_ values from †*S. macrota*. Based on climate simulations in Zhu et al. (2020), 3× and 6× preindustrial CO_2_ levels result in average sea surface temperatures for the mixed layer of 14–16°C and 16–18°C, respectively (Figures 5a and 5d; Zhu et al., 2020) and mean δ^18^O_H2O_ values for Seymour Island of −1.29‰ and −2.54‰, respectively (Figures 5b and 5e). These results are substantially lower than δ^18^O_H2O_ estimates reported in Douglas et al. (2014) using Δ_47_ from fossil bivalve shells collected from the La Meseta Fm.; however, a revised Δ_47_ calibration from Kelson et al. (2017) applied to the Douglas et al. (2014) results estimate δ^18^O_H2O_ values similar to these results from Zhu et al. (2020) from the same fossil bivalve data (see explanation above in section 5.2). Finally, we leverage the isotope‐enabled climate simulation results to compare between estimated δ^18^O_PO4_^*^ and empirical δ^18^O_PO4_ values. The †*S. macrota* teeth in this study resulted in an intermediate isotopic composition (mean δ^18^O_PO4_ values = 21.6‰ ± 1.6‰; Figure 4a) to those predicted for 3× and 6× preindustrial CO_2_ levels (23.0 ± 0.8‰ and 21.1 ± 1.0‰, respectively; Figures 5c and 5f). This range is within the estimates from an investigation of weathering and the Atlantic meridonial overturning circulation (Elsworth et al., 2017) as well as a study relating surface water phosphate concentrations to carbon isotope systematics in haptophyte algae (Bijl et al., 2010).

In addition, it should be noted that the δ^18^O_PO4_ variation in †*S. macrota* teeth reflects the range in seasonal variation of temperature, δ^18^O_H2O_, and δ^18^O_PO4_^*^ values as estimated from Zhu et al. (2020) (Figure 4), but also exceeds the model variability since variation in these variables is aggregated for millions of years within a TELM. Further, the empirical data are from a relatively large, marine vertebrate that likely moves through the mixed layer of the surface water column both in depth and latitude/longitude, even if †*S. macrota* did not migrate. The agreement of δ^18^O_PO4_ values from climate simulation and empirical geochemical results suggest that sea surface temperatures at Seymour Island were possibly much warmer than previously determined (Table S4 and references therein). During the depositional time of the La Meseta Fm., it is possible that †*S. macrota* could inhabit waters near Seymour Island throughout the year or with limited seasonal migration given the correspondence in seasonal variation (Figure 4b) and expected δ^18^O_PO4_^*^ values between the isotope‐enabled climate simulation (Figures 5c and 5f) and measured δ^18^O_PO4_ values from †*S. macrota*. In this scenario where †*S. macrota* has minimal seasonal migration, the high abundance during TELMs 2–5, then rapid decline in TELM 6 and 7, suggests that the rate of environmental change (i.e., water temperature or Drake Passage Opening) exceeded the ability of †*S. macrota* to adapt or cope.

### 5.4 Environmental Reconstruction: Paleoceanography

Despite stable temperature trends based on †*S. macrota* δ^18^O_PO4_ values, preliminary neodymium isotope analyses reveal a shift from −6 to −4.5 between TELM 2 (45–43 Ma) and TELM 3 (>38 Ma). Bioapatite *ε*_Nd_ is not tied to biology but imprinted from bottom water chemistry during early diagenesis before burial (Martin & Scher, 2004). Fossil fish teeth from deep‐sea sediment cores in the South Atlantic show shift from less radiogenic to more radiogenic *ε*_Nd_ values, believed to indicate the opening of Drake Passage (e.g., Scher & Martin, 2006). While deep‐sea Nd isotope records appear to provide robust reconstructions of past ocean circulation, Nd records from shallow locations are less well constrained. We are aware, for instance, of the importance of margin processes—collectively referred to as boundary exchange—that operate along margins, and which regulate the exchange of Nd between seawater and particles/sediment (e.g., Lacan & Jeandel, 2005), as well as the implications of pore‐fluid control on seawater Nd isotope ratios (Haley et al., 2017). However, the influence of boundary exchange and bottom‐up pore‐fluid Nd flux over tectonic time scales on Nd isotope records is poorly constrained (i.e., Wilson et al., 2013).

It is clear from previous work that changes in terrigenous inputs are important for shallow water Nd isotope records. While the shift to radiogenic *ε*_Nd_ values could be explained by an increase in the weathering flux from the Antarctic Peninsula, this explanation is unlikely during a phase of global cooling, which tends to suppress weathering rates. On the other hand, the regional increase in *ε*_Nd_ values, as constrained by the deep‐sea sediment records (Scher & Martin, 2006), is nearly the same magnitude of the TELM 2 to TELM 3/4 shift observed in this study. Based on the updated ages for the TELMs, we tentatively attribute the shift in *ε*_Nd_ values from sand tiger shark teeth to the early opening of Drake Passage, permitting Pacific waters into the south Atlantic where they would increase the *ε*_Nd_ value of waters in the Weddell Gyre.

While the number of *ε*_Nd_ values measured from sand tiger shark teeth in this study is few (*n* = 4), they indicate increasing radiogenic ε_Nd_ values from TELMs 2–4 (Figure 4c), which mirror the patterns observed from deep‐sea sites (IODP sites 689 and 1090) following the Drake Passage Opening (Scher & Martin, 2006). These preliminary *ε*_Nd_ results indicate Pacific inputs into the South Atlantic during TELM 2, which is ~45–43 Ma (Amenábar et al., 2020; Douglas et al., 2014). However, the relationship between TELMs 2 and 3 is complex due to fossiliferous lenses of TELM 3 being deposited as erosional surface within TELM 2 (see description in Geologic Setting). While there is some ambiguity in absolute age, the *ε*_Nd_ values from fossil shark teeth indicate the possible earlier detection of Pacific inputs at Seymour Island than the Bartonian (~41 Ma), which is the previously hypothesized period based on well‐dated deep ocean core sediments (Scher & Martin, 2006). It should be noted that our *ε*_Nd_ results cannot contribute to the potential reconstruction of Drake Passage bathymetry (i.e., extent or depth of opening), which could be probed with a *ε*_Nd_ enabled global climate model. The Nd isotope values of shark teeth from the La Meseta Fm. support changes in oceanographic conditions associated with the Drake Passage opening, although the size distribution and oxygen isotope values of the shark teeth indicate minimal paleoecological and paleoenvironmental shifts during the Middle Eocene.

## 6 Conclusion

Sharks are top predators in marine food webs, and as ectotherms, they are sensitive to environmental conditions and climate change (Paaijmans et al., 2013). Given the evidence of modern cascading effects in marine ecosystems, understanding how shark species adapt to and cope with past climate change over geologic time is critical. In the La Meseta Fm. on Seymour Island, changes in the community assemblage of sharks are attributed to changing environmental conditions during the Eocene, which have been associated with the Drake Passage opening. In this study, we consider ecological and environmental factors to interpret the paleobiological and geochemical results from †*S. macrota* teeth, a dominant taxon found throughout La Meseta Fm. TELMs 2–5 (Figure 2). This interdisciplinary approach allows us to frame our results within a larger context of shark paleoecology and climate dynamics. Fossil †*S. macrota* teeth indicate a large variation in body size but little temporal variation among TELMs (Figure 3a). Additionally, there is minimal environmental variation between TELMs based on δ^18^O_PO4_ values (Figures 3b and 4a), which suggests relatively stable ecological and environmental conditions. However, ε_Nd_ results from shark teeth in this study indicate increasing radiogenic values suggesting inputs of Pacific Ocean waters as early as TELM 2 (Figure 4c). Although the absolute timing for the La Meseta Fm. remains enigmatic, the *ε*_Nd_ value of the †*S. macrota* tooth from TELM 2 (45–43 Mya; Amenábar et al., 2020; Douglas et al., 2014) is the earliest geochemical evidence of the Drake Passage opening, as previous studies from IODP sites 689 and 1090 indicated a Bartonian age (~41 Mya; Scher & Martin, 2006). The proximity of the La Meseta Fm. to the Drake Passage likely enhances the effect of radiogenic Nd inputs from the Pacific Basin through an early Drake Passage as depicted in tectonic reconstructions (Lagabrielle et al., 2009; Livermore et al., 2007).

While the overall pattern in our study was not surprising for this time and region, we were perplexed with the relatively low δ^18^O_PO4_ values from †*S. macrota* teeth, which indicate higher temperatures than reported from other geochemical analyses or climate simulations. Two hypotheses to reconcile this difference are (1) a transition in †*S. macrota* paleoecology or (2) a shift in our understanding of the La Meseta Fm. paleoclimate. In our first scenario, †*S. macrota* would have adjusted to the effects of changing environmental conditions through seasonal migration to warmer, temperate waters similar to its modern analog, *C. taurus* (Cunningham, 2000; Kim et al., 2014). Our second scenario of stable, warm waters at Seymour Island is supported by climate simulation results from an isotope‐enabled model with forward modeling of expected δ^18^O_PO4_^*^ values, given 3× and 6× preindustrial CO_2_ levels (Zhu et al., 2020; Figures 5c and 5d). The coherence between measured δ^18^O_PO4_ values from fossil teeth (Figure 4a) and expected δ^18^O_PO4_^*^ values (Figures 5c and 5f) given ocean‐atmosphere dynamics in Zhu et al. (2020) suggests that †*S. macrota* could inhabit the waters near Seymour Island throughout the year with limited to no seasonal migration. This explanation involves acceptance of substantially warmer temperatures at high latitudes than temperatures previously reported by many global climate simulations and geochemical climate indicators. Fossil shark teeth are often featured in either paleontological or geochemical studies; here, we demonstrate how combining these approaches can leverage richer ecological and environmental understanding, especially as marine ecosystems responded to changing climate.

## Supporting information

Supporting Information S1

Data Set S1

## Data Availability

All data and model results can be found online (https://doi.org/10.6071/M34T1Z).
