# Supplementary material for: Probing the Ecology and Climate of the Eocene Southern Ocean With Sand Tiger Sharks Striatolamia macrota
Source: Paleoceanogr Paleoclimatol. 2020 Dec 8;35(12):e2020PA003997. doi: 10.1029/2020PA003997 (PMC8246854; doi:10.1029/2020PA003997)
Supplement: Supplementary file 1 — Supporting Information S1 [file PALO-35-0-s002.docx]

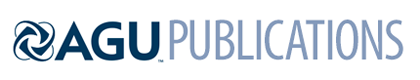


*Paleoceanography and Paleoclimatology*

Supporting Information for

**Probing the ecology and climate of the Eocene Southern Ocean with sand tiger sharks *Striatolamia macrota***

**Sora L. Kim^1,2*^, Sarah S. Zeichner^1,3*^, Albert S. Colman^1,4^, Howie D. Scher^5^, Jürgen Kriwet^6^, Thomas Mörs^7,8^, Matthew Huber^9^**

^1^ Department of Geophysical Sciences, University of Chicago, 5734 South Ellis Avenue, Chicago, IL 60637

^2‡^ Department of Life and Environmental Sciences, University of California, Merced, 5200 North Lake Rd. Merced CA 95343

^3‡^ Division of Geological and Planetary Sciences, California Institute of Technology, 1200 E California Blvd, Pasadena, CA 91125

^4‡^ Department of Earth, Environment, and Planetary Sciences, Rice University, MS126, 6100 Main St., Houston, TX 77005

^5^ Department of Earth, Ocean, and Environment, University of South Carolina

^6^ University of Vienna, Department of Palaeontology, Althanstr. 14, 1090 Vienna, Austria

^7^ Department of Palaeobiology, Swedish Museum of Natural History, P.O. Box 50007, SE-104 05 Stockholm, Sweden

^8^ Bolin Centre for Climate Research, Stockholm University, Stockholm, Sweden

^9^ Department of Earth, Atmosphere, and Planetary Sciences, Purdue University

^*^ SLK and SSZ contributed equally to this paper

^‡^ Current addresses for these authors

Corresponding author: Sora Kim ([skim380@ucmerced.edu](mailto:skim380@ucmerced.edu))

**Contents of this file**

Dataset DS1

Tables S2 to S4

**Additional Supporting Information (Files uploaded separately)**

Caption for Dataset

DS1: Anterior tooth crown heights (ATCH) measured for †*Striatolamia macrota* from La Meseta Formation separated by TELM. In addition, total length and transformed ATCH from modern sand tiger sharks from Delaware Bay are also included.

Captions for Tables

S1: Specimens with anterior tooth crown height, oxygen isotope composition, temperature, and notes.

S2: Temperature estimates using calibration curve from Kelson et al. (2017) on D_47_ values from Douglas et al. (2014).

S3: Empirical datasets and model simulations of Eocene Antarctic climate to date.

**Introduction**

Tooth crown height is associated with shark body length. We provide dataset DS1 as a .csv file with tooth crown height measurements of upper and lower anterior teeth (A1, a1, A2, and a2). The data were collected by SLK, SSZ, and JK on specimens from the University of California Museum of Paleontology (UCMP; Berkeley, CA, USA), Paleontological Research Institute (PRI; Ithaca, NY, USA), and Paleozoological Collections at the Swedish Museum of Natural History (NRM; Stockholm, Sweden).

The metadata and stable isotope composition of shark teeth analyzed in this study are given in Table S1. We report the specimen ID, collection, TELM, ATCH, mean and standard deviation of δ^18^O values (measured in triplicate), estimated temperature, and note teeth that may be from the symphyseal position. The sample preparation methods for precipitating bioapatite from shark enameloid follow procedures in Mine et al. (2017) and are described in the manuscript text.

We revisited the temperatures and estimated δ^18^O values of seawater from Douglas et al. (2014; Table S2). We applied the corrections from Kelson et al. (2017) to published D_47_ values (Douglas et al. 2014) to provide a new estimate of paleotemperature from bivalve shells coeval with the fossil shark teeth featured in this study.

The paleoclimate and palaeoceanography of the Eocene Antarctic has been explored by past studies with geochemical proxies and model simulations. We summarize and capture some of the context of these past studies in Table S3, taking note of Southern Ocean temperature ranges, proxy or model used, and CO_2_ levels.

Table S1. Specimens with anterior tooth crown height, oxygen isotope composition, temperature, and notes.

Table S2. Specimens with anterior tooth crown height, oxygen isotope composition, temperature, and notes.

******

Table S3. Empirical datasets and model simulations of Eocene Antarctic climate to date.

Data Set S1. Type or paste caption here (upload your dataset(s) to AGU’s journal submission site and select “Supporting Information (SI)” as the file type. Following naming convention: ds01.
